# Combining mGRASP and Optogenetics Enables High-Resolution Functional Mapping of Descending Cortical Projections

**DOI:** 10.1016/j.celrep.2018.06.076

**Published:** 2018-07-24

**Authors:** Jun Ho Song, Diana Lucaci, Ioana Calangiu, Matthew T.C. Brown, Jin Sung Park, Jinhyun Kim, Stephen G. Brickley, Paul Chadderton

**Affiliations:** 1 Department of Bioengineering and Centre for Neurotechnology, Imperial College London, London SW7 2AZ, UK; 2 Center for Functional Connectomics, Korea Institute of Science and Technology (KIST), 39-1 Hawolgokdong, Seoul 136-791, Korea; 3 Department of Life Sciences and Centre for Neurotechnology, Imperial College London, London SW7 2AZ, UK; 4 Institute of Neuroinformatics, UZH/ETH Zurich, Winterthurerstrasse 190, 8057 Zurich, Switzerland; 5 Division of Bio-Medical Science & Technology, KIST-School, Korea University of Science and Technology, 39-1 Hawolgokdong, Seoul 136-791, Korea; 6 School of Physiology, Pharmacology and Neuroscience, Biomedical Sciences Building, University of Bristol, University Walk, Bristol BS8 1TD, UK

**Keywords:** functional connectomics, synapse, corticofugal, synaptic integration

## Abstract

We have applied optogenetics and mGRASP, a light microscopy technique that labels synaptic contacts, to map the number and strength of defined corticocollicular (CC) connections. Using mGRASP, we show that CC projections form small, medium, and large synapses, and both the number and the distribution of synapse size vary among the IC regions. Using optogenetics, we show that low-frequency stimulation of CC axons expressing channelrhodopsin produces prolonged elevations of the CC miniature EPSC (mEPSC) rate. Functional analysis of CC mEPSCs reveals small-, medium-, and large-amplitude events that mirror the synaptic distributions observed with mGRASP. Our results reveal that descending ipsilateral projections dominate CC feedback via an increased number of large synaptic contacts, especially onto the soma of IC neurons. This study highlights the feasibility of combining microscopy (i.e., mGRASP) and optogenetics to reveal synaptic weighting of defined projections at the level of single neurons, enabling functional connectomic mapping in diverse neural circuits.

## Highlights

• Optogenetic axonal stimulation causes prolonged increases in quantal synaptic release
• Quantal and anatomical measures of synapse strength directly correspond
• Strength and cellular location of cortical inputs to midbrain are region specific

## Graphical Abstract

**Figure:**
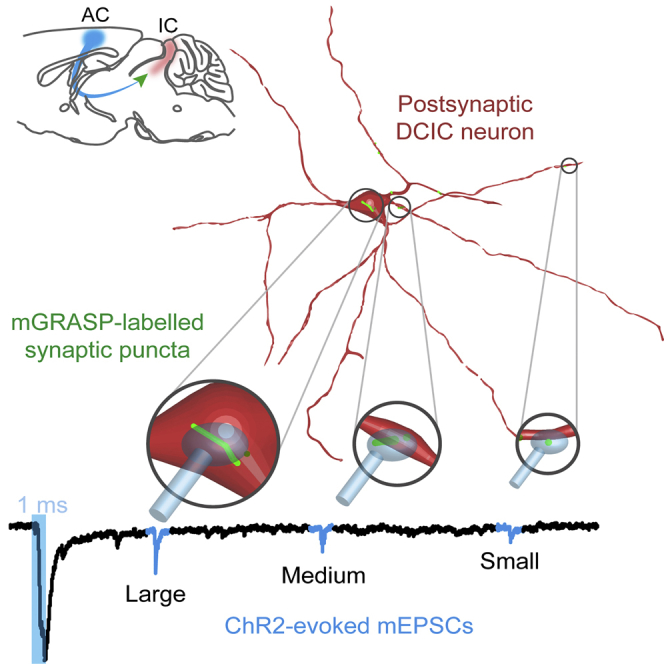

## Teaser

Song et al. combine optogenetics with anatomical mapping to determine subcellular organization of cortical input to midbrain. Functional measurements of synaptic strength correspond directly with synapse size measured using mGRASP, revealing that ipsilateral projections to dorsal inferior colliculus provide strongest descending inputs via proliferation of large somatic synapses.

## Introduction

Detailed mapping of neuronal connectivity is essential to understand the function of brain circuitry. Modern connectomics seeks to obtain a quantitative understanding of connectivity at the cellular and circuit level, gathering precise information about the number, location, and strength of synaptic inputs received onto individual neurons. Several methods have been developed to map the anatomical distribution of synapses across neurons, but these approaches do not provide information about the strength of individual connections. It is therefore difficult to infer the functional influence of connections, and circuit mapping remains incomplete. To get around this problem, we have developed a new method combining light microscopic identification and electrophysiological characterization of synaptic inputs. This approach enables both the location and the strength of defined classes of synaptic input to be quantified, providing vital functional information about the synaptic organization of neural circuitry.

Quantification of synaptic connectivity is commonly performed using electron microscopy (EM), which offers the highest resolution but is labor and data intensive and still best suited to small volumes of tissue (Saldaña et al., 1996). Light microscopy methods are more appropriate for studying large tissue volumes, albeit at a diffraction-limited resolution (Osten and Margrie, 2013). Here, we used a light microscopy technique, mammalian GFP reconstitution across synaptic partners (mGRASP) (Feng et al., 2014, Kim et al., 2011), that has the major advantage of selectively labeling only those synapses formed between neurons from defined source and target populations. To examine functional properties of mGRASP-detected synaptic contacts (e.g., connection strength), we combined the mGRASP method with an optogenetic approach to measure the magnitude of channelrhodopsin (ChR2)-evoked glutamate release from presynaptic axons in single neurons. Our results show that differences in synaptic number and size, as assayed with mGRASP, correlate exceptionally well with the postsynaptic conductance and asynchronous quantal release changes observed with optogenetics, enabling functional mapping of synaptic connectivity at the cellular and subcellular scales.

Descending projections from higher cortical regions are a prominent feature of all sensory systems. Here we demonstrate the fidelity of this approach by quantifying synaptic connectivity of descending cortical projections from the auditory cortex (AC) onto neurons of the inferior colliculus (IC). Corticocollicular (CC) feedback can influence IC processing (Bajo and King, 2013, Winer and Schreiner, 2005) and shape behavior (Bajo et al., 2010, Xiong et al., 2015). Cortical feedback to the IC is non-uniform (Bajo and Moore, 2005, Bajo et al., 2007, Coleman and Clerici, 1987, Winer et al., 1998), with the dorsal cortex of the inferior colliculus (DCIC) receiving the densest input from the AC. CC projections to this region are also bilateral, but the functional contributions of ipsi- and contralateral AC feedback remain unclear. Peters’s rule (Peters and Feldman, 1976) predicts that the differences in CC projection density will result in proportional differences in synaptic drive to IC regions. However, synaptic density does not always correlate with axon density (Druckmann et al., 2014, Kasthuri et al., 2015, Mishchenko et al., 2010), and the micro-organization of this projection remains poorly understood (Stebbings et al., 2014); we lack information about the organization of CC synaptic inputs received by individual IC neurons. Here we demonstrate the feasibility of combining optogenetics and mGRASP for the study of this CC feedback to allow differences in axonal density to be related to variability in synaptic weighting onto individual IC neurons. More broadly, we introduce a powerful approach for quantifying the functional characteristics of defined projection pathways throughout the brain.

## Results

### Density of CC Projections to Different Regions of the Mouse IC

**Figure 1:**
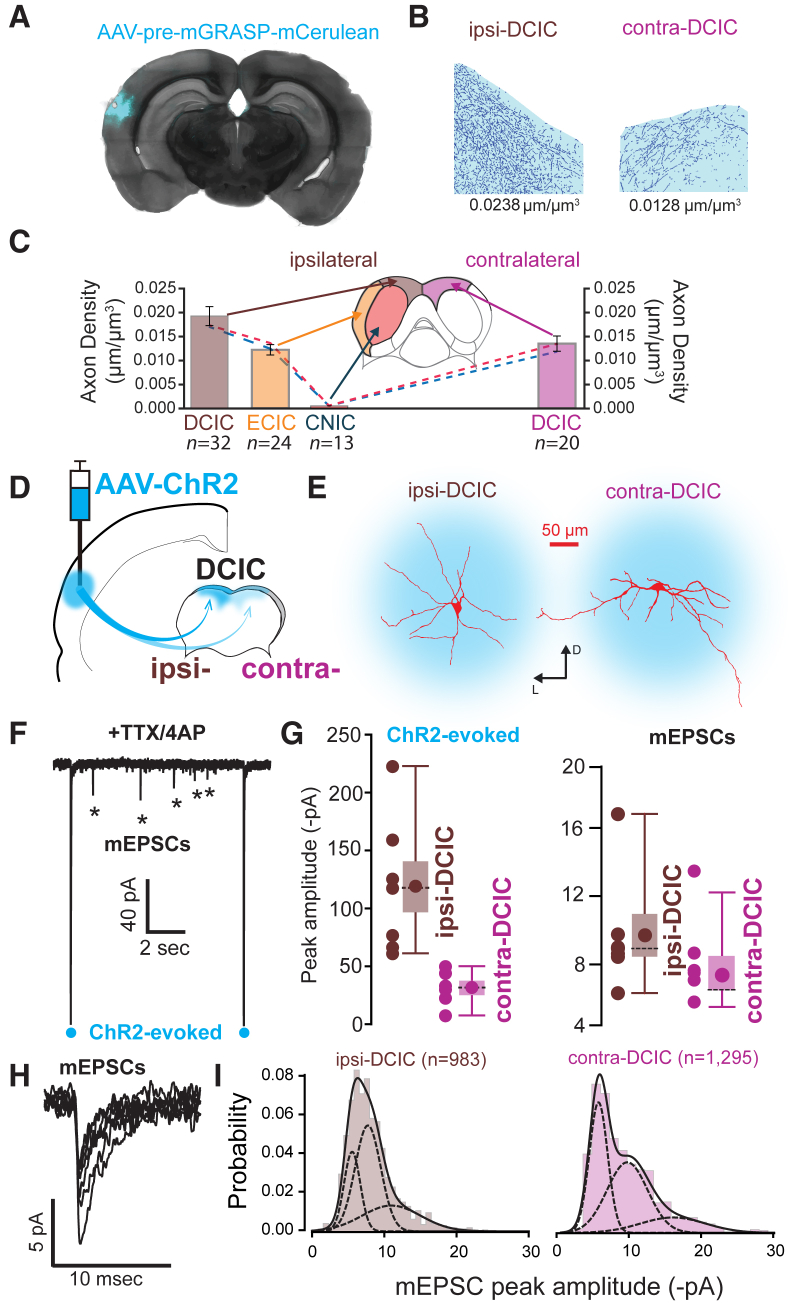
Anatomical and Functional Organization of Unilateral CC Projections to IC (A) Coronal brain section showing the location of the pre-mGRASP-mCerulean labeling of auditory cortex (AC; blue). (B) Representative images from automated detection of mCerulean-labeled axons in the contralateral dorsal cortex of the inferior colliculus (contra-DCIC) and ipsilateral dorsal cortex of the inferior colliculus (ipsi-DCIC). Values correspond to overall axonal density in these slices. (C) Axon density measurements from IC subregions (mean ± SEM). Average data were obtained from multiple brain volumes imaged from N = 4 mice. Dashed lines show the trends from paired data in N = 2 of these mice. (D) Schematic showing the injection site for adeno-associated virus (AAV) vectors used to target channelrhodopsin (ChR2) for optogenetic experiments in the contra-DCIC and ipsi-DCIC. (E) Two fully reconstructed IC neurons taken from contra-DCIC and ipsi-DCIC demonstrated the area of light stimulation used in the acute slice preparation. (F) A continuous current trace obtained during a voltage-clamp experiment from an ipsi-DCIC neuron in the presence of TTX and 4-AP. The blue circles mark the timing of 1 ms light pulses delivered through the imaging objective. Asterisks indicate mEPSCs. (G) Scatter- and box- and whisker plots comparing peak amplitudes of ChR2-evoked EPSCs and mEPSCs recorded in contra-DCIC and ipsi-DCIC. Each circle in the scatterplot is the average peak amplitude calculated in one neuron. The circle on the boxplot is the mean value obtained from all cells, the horizontal line is the median, the box denotes the SEM, and the whisker is the 5% and 95% confidence intervals. (H) Superimposed mEPSCs from the recording shown in (F). (I) Histograms constructed from mEPSC peak amplitude data obtained from contra-DCIC and ipsi-DCIC recordings illustrating the probability density function for mEPSC sizes in these regions. The solid line is the summation of the three Gaussian functions shown with dashed lines.

We first quantified the density of CC axons within subregions of the mouse IC. Taking advantage of predominant axonal expression of pre-mGRASP (Feng et al., 2014, Kim et al., 2011), we injected recombinant adeno-associated virus (rAAV) expressing mCerulean-fused pre-mGRASP into the AC and measured the density of AC axons in ipsi- and contralateral subregions of the IC, i.e., DCIC, external cortex of the inferior colliculus (ECIC), and central nucleus of the inferior colliculus (CNIC) (Figures 1A–1C). The densest of these projections were consistently observed in ipsilateral DCIC. Elsewhere in the ipsilateral hemisphere, a moderate mCerulean signal was observed in ECIC, with only sparse signals in CNIC. In the opposing hemisphere, a moderate signal was observed in contralateral DCIC with little, if any, mCerulean signal in CNIC and ECIC. To quantify CC projections in each IC region, axonal densities were determined using an automated detection algorithm (Figure 1B) (see Experimental Procedures). Automated analysis of IC volumes matched our subjective assessment of fluorescent intensity: for ipsilateral CC projections, the DCIC received the highest average axonal density (0.0194 ± 0.0020 μm/μm^3^, n = 32), followed by the ECIC (0.0123 ± 0.0011 μm/μm^3^, n = 24), with the lowest average axonal density (0.0005 ± 0.0001 μm/μm^3^, n = 13) in the CNIC. For contralateral CC projections, the only detectable signal was found in the DCIC (0.0136 ± 0.0016 μm/μm^3^, n = 20). Overall, our quantitative mapping of CC projections (Figure 1C) gave comparable results to previous measurements (Bajo and Moore, 2005, Winer et al., 1998).

### CC Synaptic Weighting within Different Regions of the Mouse IC

We next compared the relationship between axonal density and strength of CC synaptic input received by individual IC neurons. Unilateral stereotaxic injections of the AAV-ChR2 virus into the AC was used to express ChR2 in CC axons within acute slices of mouse IC (Figure 1D). *In vitro* whole-cell voltage-clamp recordings were made from IC neurons using blue light pulses to activate transduced CC fibers and evoke excitatory postsynaptic currents (EPSCs). For our functional studies, we concentrated on contralateral dorsal cortex of the inferior colliculus (contra-DCIC) and ipsilateral dorsal cortex of the inferior colliculus (ipsi-DCIC), because these regions were most easily identifiable in the acute slice preparation. The membrane capacitances of neurons recorded in the two regions were similar (38.4 ± 5.4 pF, n = 7, for ipsi-DCIC neurons compared to 39.5 ± 3.2 pF, n = 6, for contra-DCIC neurons recorded under identical pharmacological conditions), indicating that neuronal morphologies were similar in the two regions. This was confirmed post hoc from morphological measurements obtained via confocal imaging, in which the mean surface area was measured to be 2,386.4 ± 134.5 μm^2^ (n = 32) for ipsi-DCIC neurons and 2,575.3 ± 185.2 μm^2^ (n = 24) for contra-DCIC neurons.

For optogenetic stimulation, an illumination spot of sufficient size (31,415 μm^2^) was used to ensure that nearly all the somatodendritic axis of recorded neurons was illuminated during light stimulation (Figure 1E). In neuronal recordings made in regular artificial cerebrospinal fluid, blue light pulses delivered through the imaging objective resulted in ChR2-evoked responses consisting of multiple postsynaptic current transients occurring at both short and long latencies (data not shown). Therefore, to avoid indirect polysynaptic activation of synaptic release onto DCIC neurons, the voltage-gated sodium channel blocker tetrodotoxin (TTX; 1 μM) was added to the slice preparation, along with the voltage-gated potassium channel blocker 4-aminopyridine (4-AP; 200 μM) (Figure 1F). ChR2-evoked responses recorded in TTX/4-AP were smaller than those observed in control conditions and were characterized by a monotonically rising EPSC that decayed with a slower exponential decay, consistent with a direct monosynaptic activation of CC axons only. We expected that the density of transduced CC axons would correlate with the relative magnitude of evoked EPSCs in ipsi- and contra-DCIC. The average peak amplitude of ChR2-evoked postsynaptic responses (the average of at least eight responses in each cell recorded from 2 mice) (Figure 1G) was greater (p = 0.0006 using a Mann-Whitney test) in the ipsi-DCIC (−118.3 ± 2.7 pA, n = 6) compared to the contra-DCIC (−31.05 ± 2.5 pA, n = 7), highlighting that CC axon density is an important indicator of projection strength in single neurons.

In addition to evoking large short-latency EPSCs, we observed that blue light stimulation produced a prolonged elevation in the rate of asynchronous miniature EPSCs (mEPSCs) (Figures 1F and 1H). We compared the amplitude distributions of these mEPSCs in ipsi- and contra-DCIC neurons. Although the mEPSC overall mean amplitude was not significantly smaller between ipsi- and contraregions (mEPSC peak amplitude: −9.7 ± 1.3 pA versus −8.3 ± 2.7 pA; n = 7 and 6, respectively; p = 0.94 using a Mann-Whitney test), close examination of amplitude distributions in the two locations revealed differences in the mEPSC peak amplitude distributions. All-point histograms were constructed from contra- and ipsi-DCIC mEPSC peak amplitude values (see Experimental Procedures), and these distributions were fitted with the sum of one to four Gaussians. The resulting χ^2^ values obtained from least-squares fitting were used to determine any improvement in the fit. For the fit of ipsi-DCIC data, a single Gaussian function gave a χ^2^ of 22.1 that reduced to 10.48 with two Gaussians and to 7.63 with three Gaussians. The error associated with the fit did not reduce further when a fourth Gaussian was added to the function. Analysis of fits revealed differences in the contributions of small-, medium-, and large-amplitude mEPSCs recorded in the contra-DCIC and ipsi-DCIC following ChR2 stimulation (Figure 1I). A smaller proportion of large mEPSCs was present within the contra-DCIC population, as evidenced by a reduction in the area of the third Gaussian (27% of the total area in the probability-density function for the ipsi-DCIC data versus 11% of the total area in the contra-DCIC) and an increase in the small mEPSC peak amplitudes (23% in the ipsi-DCIC population, increasing to 38% in the contra-DCIC). We also noticed a wider separation among the centers of the small, medium, and large mEPSC peaks in the contra-DCIC.

**Figure 2:**
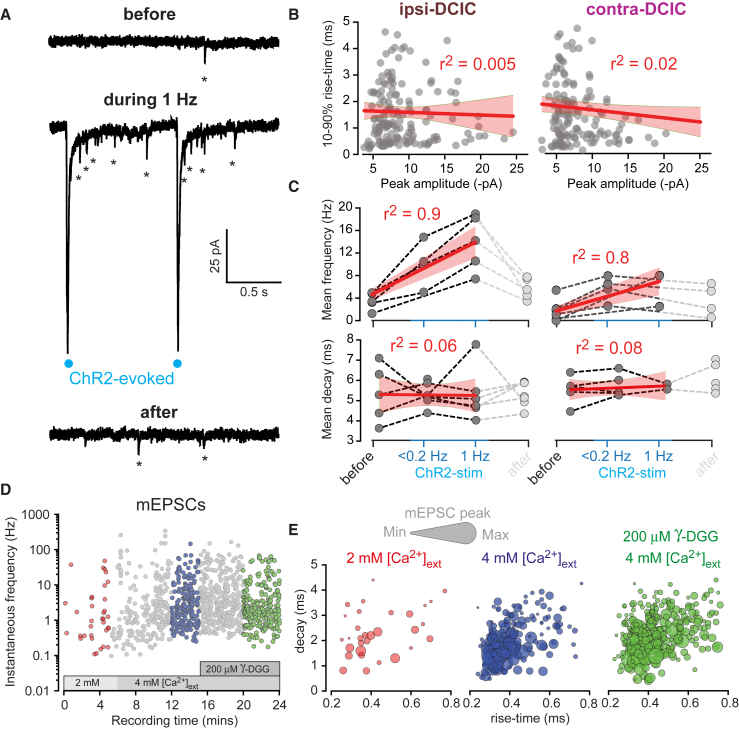
Light Stimulation of ChR2-Expressing CC Terminals Leads to a Rate-Dependent Increase in Asynchronous Quantal Release (A) Results from a typical voltage-clamp experiment recorded in the presence of TTX and 4-AP, illustrating three current traces from the same ipsi-DCIC neuron obtained before, during, and after 1 Hz stimulation of ChR2-expressing CC terminals. Blue circles mark the timing of 1 ms light pulses delivered through the imaging objective. Asterisks indicate the incidence of spontaneously occurring mEPSCs. (B) Scatterplots of mEPSC peak amplitudes against 10%–90% rise times for recordings made from IC neurons in the ipsi-DCIC and contra-DCIC. Filled gray circles are values for each mEPSC, and the solid red line is the result of a linear regression analysis of all data, with the shaded areas depicting the 5% and 95% confidence limits for these fits. The very low r^2^ values indicate no relationship between peak amplitude and rise time. (C) Scatterplots of mEPSC mean frequency recorded from IC neurons before, during, and after ChR2 stimulation. The solid red line is the result of a linear regression analysis, and the shaded areas are the 5% and 95% confidence limits for these fits. The high r^2^ values indicate that a large proportion of the data is described by a simple linear relationship. Light gray symbols are the recovery values for each IC neuron. These values were not used for the regression analysis. In addition, we were not able to analyze the frequency of mEPSCs at all stimulation frequencies in every cell. The same analysis was repeated for the weighted decay values obtained for the mEPSC populations, demonstrating little change in the decay of the mEPSCs before, during, and after optogenetic stimulation. (D) Scatterplot of mEPSC instantaneous frequency during an experiment in which the external Ca^2+^ concentration was raised and the low-affinity glutamate antagonist γ-DGG was applied. In this recording, the mEPSC frequency increased from a median value of 1.08 Hz (red symbols) to 2.43 Hz (blue symbols) in 4 mM Ca^2+^. The median mEPSC frequency did not change in 200 μM γ-DGG (green symbols). (E) Scatterplots of 10%–90% rise time and weighted decay estimates illustrating the peak amplitude of each mEPSC with different-sized bubbles. Note the similar distributions for the different epochs highlighted in (D).

Multiple peaks in mEPSC amplitude distributions could reflect the presence of distinct populations of small, medium, and large postsynaptic densities. The observed differences in the distributions of these small, medium, and large mEPSC peaks were only detectable following ChR2 stimulation. Presynaptic depolarization associated with other types of presynaptic stimulation has been previously shown to increase spontaneous transmitter release (Cummings et al., 1996). We therefore considered that a similar mechanism could be present during optical stimulation of CC axons, leading to the observed increase in quantal events in the postsynaptic IC cells. To test for this possibility, we measured mEPSC frequency before, during, and after optogenetic stimulation of contra- and ipsi-DCIC at different rates (Figure 2A). Before stimulation, the average frequency of mEPSCs was low in both ipsi- and contra-DCIC (2.7 ± 0.3 Hz, n = 6, and 3.5 ± 0.7 Hz, n = 7, for ipsi- and contra-DCIC, respectively). Following optogenetic activation, there was an increase in the frequency of mEPSCs in both regions (p = 0.0005 in the ipsi-DCIC and p = 0.002 in the contra-DCIC using a Mann-Whitney U test). The peak amplitude and 10%–90% rise time of mEPSCs detected during ChR2 stimulation was analyzed to look for the presence of multi-vesicular release that could explain differences among regions. The larger mEPSCs were not associated with slower rise times (linear regression analysis), consistent with a lack of mEPSC superimposition in these recordings (Figure 2B). In ipsi-DCIC, the frequency of mEPSCs increased to 10.1 ± 2.0 Hz when the optical stimulation rate was <0.2 Hz and increased further to 13.9 ± 2.2 Hz at an optical stimulation rate of 1 Hz. Regression analysis of the mEPSC frequency at different stimulation rates gave a slope of 4.6 ± 0.4 Hz/Hz (r^2^ = 0.89) in the ipsi-DCIC compared to 2.6 ± 0.4 Hz/Hz (r^2^ = 0.80) in the contra-DCIC that was significant in both regions (Figure 2C). The increased slope in the ipsi- versus contra-DCIC data is consistent with the hypothesis that optical stimulation causes elevated spontaneous transmitter release in ChR2-expressing axons and that a denser CC projection (Figure 1C) affords a larger number of enhanced active release sites in ipsi-DCIC. To our knowledge, this observation involving ChR2 stimulation has not been made previously and provides a novel means to isolate mEPSC events from ChR2-expressing projection fibers.

Transmitter pooling could occur during these optogenetic experiments, so we examined mEPSC decays during the different stimulation conditions (Figure 2C). For the ipsi-DCIC data, the mean decay was 5.34 ± 0.53 ms (n = 5) in control conditions, and this did not alter when mEPSC frequency was increased during <0.2 Hz stimulation (5.28 ± 0.21 ms, n = 7) or during 1 Hz stimulation (5.29 ± 0.54 ms, n = 6). This was also true for the contra-DCIC data, for which we failed to detect any effect on 10%–90% rise time, peak amplitude, or weighted decay of mEPSCs. An alternative method for examining the influence of transmitter pooling is to use a low-affinity antagonist such as gamma-D-glutamylglycine (γ-DGG) to selectively block glutamate receptors that could theoretically be activated by the lower concentration of glutamate outside of the synaptic cleft (Wadiche and Jahr, 2001). To enhance the likelihood of transmitter pooling taking place, we made recordings in raised external calcium concentrations to increase glutamate release (Figure 2D). As expected, raising external calcium increased the mEPSC frequency from 0.71 ± 0.11 Hz in 2 mM Ca^2+^ to 3.24 ± 0.57 Hz in 4 mM Ca^2+^ (n = 7), but this increased rate of glutamate release had no effect on mEPSC properties (rise, peak, or decay), and the addition of 200 μM γ-DGG did nothing to the mEPSCs recorded from the DCIC (Figure 2E). Therefore, the different mEPSC populations identified with peak amplitude distributions (Figure 1H) are likely to reflect the activation of different-sized glutamate receptor populations located at independent release sites.

### mGRASP-Based Structural Synaptic Mapping of CC

**Figure 3:**
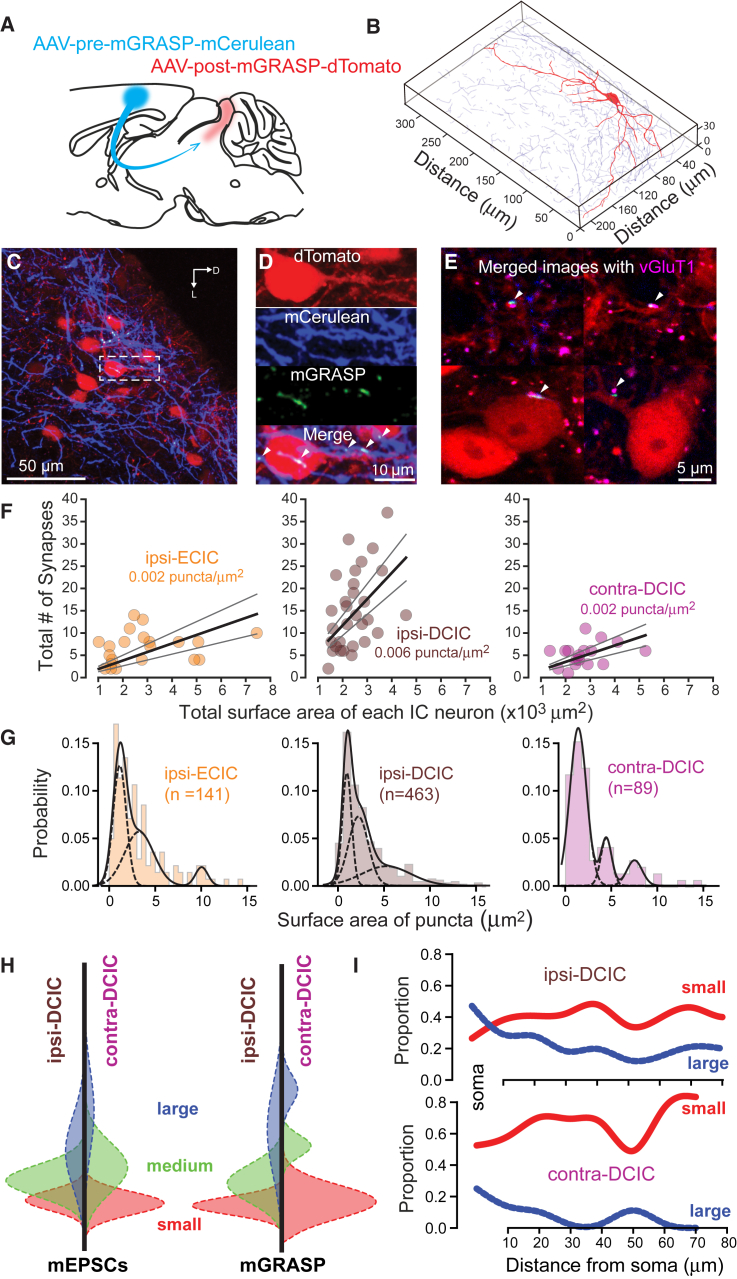
Correspondence of Anatomical and Functional Distributions of Small, Medium, and Large Synapses across the Somatodendritic Axis of Ipsi- and Contra-DCIC Neurons (A) Schematic demonstrating the unilateral injection of the pre-mGRASP viral vector into the AC and bilateral delivery of the post-mGRASP viral vector into the IC. (B) 3D reconstruction of a postsynaptic IC neuron (red) and the presynaptic mGRASP signal in the dorsal region of the contra-DCIC. (C) Exemplar confocal image from the ipsi-DCIC. Post-mGRASP-labeled IC neurons (red) are shown, along with pre-mGRASP-labeled axons (blue) projecting from the AC. The dashed box indicates the region of this image that is shown at higher magnification in (C) to identify the mGRASP signals (green). (D) Blue, red, and green channels are separated to illustrate the location of the presynaptic CC axons (blue), the postsynaptic IC neuron (red), and the GFP-labeled mGRASP signals (green). The bottom image is the merger of the three images above. (E) Four merged images similar to the bottom image in (D) but containing a purple stain for VGluT1. (F) Scatterplots showing the total number of mGRASP synapses identified on each neuron (filled circles) in relation to the surface area of each fully reconstructed neuron. The solid lines show the results of linear regression analysis for the data obtained in the ipsi-ECIC, ipsi-DCIC, and contra-DCIC. The dashed lines are the 5% and 95% confidence limits for these fits. (G) All-point histograms illustrating the probability density functions for mGRASP puncta of varying sizes within the ipsi-ECIC, ipsi-DCIC, and contra-DCIC. (H) Mirrored probability density functions of small (red), medium (green), and large (blue) synapse distribution in ipsi-DCIC (left) and contra-DCIC (right) assessed via mEPSC amplitude (left-hand plots) or mGRASP puncta surface area (right-hand plots). (I) Location of small and large synapses across the somatodendritic axis of DCIC neurons. Ipsilateral CC connectivity to the IC (ipsi-DCIC) is characterized by an increased proportion of large proximal synapses compared to contralateral CC (contra-DCIC).

Given that our optogenetic experiments indicated the existence of various synaptic populations (with small, medium, and large mEPSCs) differentially distributed across ipsi- versus contra-DCIC neurons, we used mGRASP labeling to determine whether a similar weighting in anatomically defined synaptic sites was apparent between the CC input to ipsi- and the CC input to contra-DCIC. The mGRASP technique permits identification of synaptic contacts between neurons via reconstitution of non-fluorescent GFP fragments (Druckmann et al., 2014, Kim et al., 2011). We injected pre-mGRASP-expressing rAAV (pre-mGRASP-mCerulean) into AC and post-mGRASP-expressing rAAV (post-mGRASP-2A-dTomato) into both ipsi- and contralateral IC (Figures 3A and 3B) (see Experimental Procedures). IC neurons in all regions demonstrated the red fluorescent signal of cytosolic dTomato coexpressed with the post-mGRASP component. Synaptic contacts between labeled CC axons and IC neurons were identified as spots of green fluorescence, emitted from the fully reconstituted GFP. Green spots, corresponding to synaptic contacts between AC axons and IC neurons, were observed on both soma and dendrites (Figures 3C and 3D). Antibody staining revealed mGRASP signals to be colocalized with the synaptic protein vGluT1 (Figure 3E), confirming their identity as synaptic markers.

Full 3D reconstructions of IC neurons and associated CC axons were generated (Figure 3B; Figure S1) to count and localize mGRASP-labeled synaptic contacts present on single neurons. mGRASP-labeled synaptic contacts were defined as GFP signals that were associated with both a labeled presynaptic CC axon and a fully reconstructed postsynaptic IC neuron. Consistent with measurements of CC axon density and evoked EPSC amplitude, the largest number of mGRASP-labeled synaptic contacts was found on IC neurons within the ipsi-DCIC (Figure 3F). Whereas CNIC (in both hemispheres) and contralateral ECIC were devoid of puncta, GFP signal was present in the ipsilateral ECIC and contra-DCIC, consistent with automated CC axon density measurements (Figure 1C). In ipsi-DCIC, 30 of 32 reconstructed IC neurons (94%) showed evidence of mGRASP-labeled synaptic contacts, compared with 17 of 20 neurons (85%) in contra-DCIC, and 21 of 24 neurons (87.5%) in ipsilateral ECIC. The average number of mGRASP-labeled synaptic contacts was found to be 15 ± 2 puncta/neuron for ipsi-DCIC neurons (range 3–34, n = 30), 5 ± 0.5 puncta/neuron for contra-DCIC neurons (range 1–11, n = 17), and 7 ± 0.5 puncta/neuron for external cortex of the inferior colliculus (ipsi-ECIC) neurons (range 2–14, n = 21). We next examined the relationship between neuronal surface area and number of mGRASP-labeled synaptic contacts for neurons in different IC subregions. All neuronal populations exhibited positive correlations between the number of mGRASP-labeled synaptic puncta and the neuronal surface area (Figure 3F). The slope of this relationship was greatest for ipsi-DCIC neurons (0.006 puncta/μm^2^) compared to contra-DCIC neurons (0.002 puncta/μm^2^) and ipsi-ECIC neurons (0.002 puncta/μm^2^). The clear regional differences in the densities of mGRASP-labeled synaptic contacts are consistent with densities of CC axons (Figure 1B) and ChR2-evoked mEPSC amplitudes in the different IC subregions (Figure 1G).

### Functional and Structural Correspondence of CC Synaptic Distributions

The size of the mGRASP-labeled synaptic contacts could provide a reliable method for estimating the size of the postsynaptic density. Therefore, we compared the relationship between the postsynaptic conductance changes obtained from the mEPSC distributions and the surface area distribution of individual mGRASP-labeled synaptic contacts. Using the same analytical approach as for mEPSC distributions (least-squares fitting of functions with increasing numbers of Gaussians and subsequent monitoring of the adjusted-r^2^ values for the cumulative fits), we observed that the distributions of mGRASP surface area measurements obtained from individual neurons in each IC subregion were best described by the sum of three Gaussians (Figure 3G). Again, addition of the fourth Gaussian made no difference to the adjusted-r^2^ value, and the area of the fourth peak was less than 1% of the total area. Using the three Gaussian functions contributing to the cumulative fit, we estimated that 31% of ipsi-DCIC CC synapses were made up of small puncta, 41% were from medium puncta, and 28% were from large puncta. For comparison, the probability density function generated for the contra-DCIC distribution indicated that 74% of CC synapses formed small puncta, 15% formed medium puncta, and only 11% were made up of the largest puncta. A similar lack of large puncta was present for the ipsi-ECIC data, with 46.5% small puncta, 46.5% medium puncta, and only 7% of the population provided by the largest puncta. Therefore, the mGRASP signal demonstrated differences in the distribution of small, medium, and large synapses in the different IC subregions. The mEPSC amplitude distributions (Figure 1I) for ipsi-DCIC and contra-DCIC neurons showed similar differences in the area of the three Gaussians (Figure 3H). From the area of each Gaussian in the probability density function, we estimated that the smallest mEPSCs contributed 38% of the entire population in the contra-DCIC, whereas this portion of the population was only 23% in the ipsi-DCIC. This increased contribution from small synapses is seen for both the mEPSC and the mGRASP data (Figure 3H).

Given the striking accuracy of the mGRASP technique in predicting differences in the mEPSC data, we went on to exploit the mGRASP data to define the distribution of synapses across the IC neuronal compartments. Cutoff values were defined from the probability density functions constructed from the mGRASP data to estimate the proportion of small, medium, and large synapses across the IC neuron soma and dendrites (Figure 3I). These plots describe how the larger synapses are more prominent in the soma of ipsi-DCIC neurons (n = 30), whereas the smaller synapses are a more dominant feature of all analyzed compartments in the contra-DCIC neurons (n = 17). Therefore, neurons in ipsi-DCIC preferentially receive a larger proportion of large, somatic targeting synapses that contribute relatively higher-amplitude CC synaptic currents in these cells.

## Discussion

In the present study, we demonstrate that the subcellular location and strength of synaptic contacts can be revealed using a combination of mGRASP and optogenetics. In summary, the ipsilateral CC projection generated mGRASP-labeled synaptic contacts within the DCIC and ECIC, but not the CNIC. The only contralateral location exhibiting detectable mGRASP-labeled synaptic contacts was the DCIC. Therefore, individual DCIC neurons receive substantial synaptic input from both hemispheres of the AC. Using both functional approaches (i.e., optogenetics and electrophysiology, measuring mEPSC amplitude) and anatomical approaches (i.e., mGRASP, measuring synapse surface area), we demonstrate that CC inputs onto IC cells can be separated into three distinct groups: small, medium, and large synapses. Distributions among these groups were similar for functional and anatomical data with respect to both ipsilateral and contralateral CC projections (Figure 3H). Our results demonstrate that the increased synaptic weighting of the ipsilateral CC projection is due to a greater proportion of large synapses, and this is particularly obvious in ipsilateral CC synapses found on the soma of DCIC neurons.

### Synaptic Organization of Auditory Cortical Feedback to IC

The strength, location, and number of CC synaptic inputs onto individual neurons will influence the potency of AC feedback upon sensory encoding within IC. Previous macroscopic anatomical studies have inferred connection strength between the AC and the IC by examining CC axon density (Bajo and Moore, 2005, Bajo et al., 2007, Winer et al., 1998) and applying Peters’s rule (Peters and Feldman, 1976). Our results using mGRASP are consistent with the view that CC input to the CNIC is negligible and the major CC input is to the DCIC, with greater ipsilateral weighting. The use of genetically encoded calcium indicators to assay action potential firing in CC axon terminals in the IC has functionally demonstrated stronger CC input to the ipsi-DCIC (Barnstedt et al., 2015). High-resolution EM has demonstrated a similar amount of CC input to the distal dendrites of IC neurons in all IC subregions (Saldaña et al., 1996); here our results indicate an additional weighting of large somatic synapses from ipsilateral CC projections. Previously, the expression of the vesicular glutamate transporter vGluT1in neocortical pyramidal cells (Fremeau et al., 2001, Fujiyama et al., 2001) was used to identify the location of CC inputs on IC neurons. These studies reported that vGluT1-immunolabeled puncta were rarely observed on the soma and were predominantly found on dendrites (Altschuler et al., 2008). However, the fidelity of our anatomical assay (mGRASP-generated synaptic surface area distributions) is reinforced by the correspondence with functional measurements of mEPSC amplitude distributions. Our data support the view that cortical influence is strongest from the ipsilateral hemisphere because of an increased fraction of large, somatically located synapses received by DCIC neurons. Differences in the strength of the bilateral CC projections to the DCIC may facilitate neural computations involved in sound localization, such as interaural timing or intensity differences (Chase and Young, 2008).

### Combining Optogenetics and mGRASP for Functional Projection Mapping

Our results demonstrate the utility of mGRASP as an approach to infer functional connectivity among regions of the brain. The remarkable correspondence between mEPSC peak amplitude distributions and mGRASP puncta is consistent with quantitative immunolabeling studies that demonstrated how quantal size is determined by the number of ligand-gated ion channel proteins found in the postsynaptic density of inhibitory synapses (Lim et al., 1999, Nusser et al., 1997). Similar EM analysis at corticothalamic synapses demonstrated how quantal amplitude reflected the number of glutamate receptors, explaining the larger descending synaptic input to the reticular compared to thalamic relay neurons (Golshani et al., 2001). Here we establish the suitability of mGRASP for quantitative functional circuit mapping. Given a uniform receptor density, it is not surprising that mEPSC peak amplitude distributions compare so favorably with the surface area distributions obtained using the mGRASP technique. Our optogenetic method exploits the selective increase in release probability following stimulation of ChR2-positive axons. It therefore relies on the ability to activate all available release sites onto the neuron being studied and further depends on the ability of the patch-clamp technique to accurately estimate receptor number without introducing space-clamp artifacts. Future studies also need to establish whether all preparations are suitable for the mGRASP approach, because it cannot be assumed that all postsynaptic sites have equal receptor density (Tarusawa et al., 2009). However, the striking advantage of this combination of techniques is that we can now identify the synaptic weighting of defined inputs to given neuronal populations, without data- and time-intensive EM methods. The application of optogenetics and mGRASP thus provides a powerful approach for quantifying the characteristics of defined projections throughout the brain, enabling functional connectomic mapping of diverse neuronal pathways.

## Experimental Procedures

### Surgical Procedures and Animal Handling

The care and experimental manipulation of animals was undertaken in accordance with institutional guidelines and the United Kingdom Animals (Scientific Procedures) Act 1986. C57BL/6 mice (female, 6–12 weeks old, N = 8) were anaesthetized via intraperitoneal (i.p.) administration of a fentanyl, midazolam, and medetomidine mixture (0.05, 5.0, and 0.5 mg/kg). Following induction of anesthesia, mice were placed in a stereotaxic apparatus and maintained at physiological temperature via a feedback-controlled heat pad (FHC, USA). A midline incision was made, and tissue was carefully cleared from the cranium. Bregma and lambda fissures were identified under stereoscope, and the head was re-adjusted in the stereotaxic frame so that the difference between the dorsoventral coordinates of bregma and lambda was less than 10 μm. Two craniotomies—one above the right AC and the other above the bilateral IC—were made to enable delivery of rAAV. Injection pipettes were carefully inserted into target brain regions, and exceptional care was taken to avoid damage to passing blood vessels. In the AC, rAAV2/1-expressing pre-mGRASP (AAV-CAG-pre-mGRASP-mCerulean) or ChR2 (AAV-hSyn-hChR2(H134R)-EYFP) constructs were delivered at a speed of 25 nL/min using a Hamilton syringe. The stereotaxic coordinates used were action potential (AP) 1.50, mediolateral (ML) −0.40, and dorsoventral (DV) 2.00 at a 0° angle. To ensure transduction of the entire AC, 2–3 separate injections (100–200 nL) were made per preparation spaced by 150–200 μm in the anteroposterior axis, using viral serotype and promoter combinations that drove dense expression throughout all layers of the cortex (Feng et al., 2014). In the IC, target subregions—the ipsilateral dorsal cortex (DC), external cortex (EC), and central nucleus (CN) and contralateral DC—were injected with 25–50 nL of rAAV2/1-expressing post-mGRASP (AAV-CAG-post-mGRASP-2A-dTomato) at a speed of 25 nL/min. After each injection, pipettes were left undisturbed for 10 min to allow the viral solution to be absorbed by the tissue; thereafter, pipettes were removed slowly. After injection, craniotomies were sealed with Kwik-Cast (World Precision Instruments, USA), and the scalp was sutured with glue (Histoacryl, Braun, USA) or nonabsorbable nylon sutures (Ethilon, Ethicon, USA). Immediately after suturing, anesthesia was antagonized via injection of a naloxone, flumazenil, and atipamezole mixture (1.2, 0.5, and 2.5 mg/kg, i.p.). Postoperative analgesia was provided via eutectic mixture of local anesthetics (EMLA; 2.5% lidocaine and prilocaine) application to the sutured skin. Mice were placed in a heated recovery box under observation until they fully woke from anesthesia; thereafter, animals were returned to their home cage.

### Preparation and Imaging of mGRASP-Labeled Tissue

2–4 weeks after surgery, mGRASP-transduced mice were deeply anaesthetized with ketamine (100 mg/kg, i.p.) and were perfused via the ascending aorta with 15 mL of 0.1 M phosphate buffer (PB) (pH 7.4) solution to followed by 25–30 mL of 4% w/v paraformaldehyde (PFA, Sigma-Aldrich) in distilled water to avoid formation of salt crystals that can produce fluorescence artifacts. Brains were removed and left in 4% w/v PFA solution for 4 hr at 4°C. After fixation, brains were washed three times, 15 min per wash, in 0.1 M PB (pH 7.4) solution on a shaker and then embedded in 5% w/v agarose in distilled water. Brain tissue was sectioned with a vibratome (VT1000S; Leica, Germany) to produce 100 μm thick slices. Individual slices were washed again with 0.1 M PB (pH 7.4) solution to avoid salt crystallization and were mounted onto glass slides. Slices were treated with mounting media (Vectashield H-1000, USA) and covered with a glass coverslip.

To validate mGRASP as a synapse detector, we performed immunostaining with anti-vGluT1 and anti-GFP in infected coronal brain slices (50 to ∼100 μm), as previously described (Druckmann et al., 2014). The following antibodies were used: rabbit anti-vGluT1 (Synaptic Systems, 1:1,000, RRID: AB_887875); chicken anti-GFP (Abcam, 1:500, RRID: AB_300798); and Alexa 488- and Alexa 633-conjugated secondary antibodies (Invitrogen, 1:1,000, RRID: AB_2534096 and RRID: AB_2535732, respectively).

IC slices were imaged using a confocal microscope (SP5; Leica, Germany). Appropriate excitation wavelengths were used for different fluorophores: 458 nm for mCerulean (pre-mGRASP-labeled axons), 488 nm for GFP (mGRASP-labeled synapses), and 561 nm for dTomato (post-mGRASP-labeled IC postsynaptic neurons). The IC subregion that a neuron belonged to was determined using the mouse brain atlas (Franklin and Paxinos, 2012) and apparent axon density; neurons located in ambiguous or border regions were excluded. Axons, synapses, and postsynaptic IC neurons were imaged separately with carefully adjusted spectral detectors to avoid any potential bleed-through. The number of optical sections varied depending on the dimension of each neuron, with an optical section thickness of 0.5–0.6 μm. Based upon the point spread function of the imaging system, we can achieve a maximum resolution of 0.4 μm in the lateral axis and 0.8 μm in depth.

### Anatomical Tracing

Raw confocal images were first preprocessed with Fiji to adjust the contrast and despeckle (Schindelin et al., 2012). Thereafter, individual IC neurons (32 neurons in ipsi-DCIC, 24 neurons in ipsi-ECIC, 13 neurons in the ipsilateral CNIC, and 20 neurons in the contra-DCIC) and synaptic contacts were manually traced in 3D with neuTube (Feng et al., 2015). To avoid false-positive counting of synapses, we visually confirmed that the synapses observed were made on postsynaptic IC neurons with AC axons passing nearby. Traced neurons and synapses were saved in .swf formats.

To analyze axonal densities in IC, we created a semi-automatic tracing algorithm, trace-3D, implemented in MATLAB. The method is based on vectorial tracking (Al-Kofahi et al., 2002) and has two main stages: first, starting points (seeds) are generated from 3D confocal stacks to identify labeled axons, and then the local properties of the image at each seed are explored in a recursive manner to complete tracing.

Seed-point selection is done through a five-step algorithm. In step 1, 3D datasets are projected onto the x-y plane using the maximum intensity across the z dimension. In step 2, user-defined upper and lower thresholds are set for binning the 2D image—this is the only step that requires user interaction. In step 3, a distance transform algorithm is performed, which consists of scoring each foreground pixel according to how far it is from the nearest background pixel. Boundary pixels will have a value of 1, and pixels on the axonal median axis will have the highest local value. In step 4, local maxima are located within windows of N × N pixels to generate the x, y coordinate of the seeds. For this dataset, we used N = 5 pixels to ensure complete coverage of the torturous and highly compact axonal segments found in ipsi-DCIC slices. In step 5, for each pair of x, y coordinates, the algorithm looks across all z coordinates and chooses the one with the highest intensity pixel.

Following seed selection, the second part of the tracing consists of a 3D vectorial tracking algorithm that is done automatically through a three-step procedure. In step 1, the 3D dataset, *I*, is convolved with a Gaussian filter, *G*_0_, to make it (almost) differentiable (*G* = *G*_0_^∗^ *I*). This step is done using Statistical Parameter Mapping (SPM) software that is especially designed for analysis of large brain-imaging datasets. The width of the filter (sigma) governs the fidelity of this stage: using a higher scale value sigma for the Gaussian blurring leads to an increase in tracing performance by averaging out the noise found in data generated through confocal microscopy. However, tortuous axonal segments that are close to one another would end up being merged, without the possibility of being separated. We found the best fit for our dataset to be a value of σ = 5 pixels. In step 2, the Hessian matrix (second-order partial derivatives of *G*) of a voxel (9 × 9 × 9 pixels to capture the whole volume of the axon and the surrounding background) is computed to extract the orthonormal eigenvector basis (with *λ*_1_, *λ*_2_, and *λ*_3_ eigenvalues). By comparing the magnitudes of the eigenvalues, tubular-like geometrical structures are detected that fulfill the property *λ*_2_ ≈ *λ*_3_ ≪ 0, *λ*_1_ ≈ 0 (Orłowski and Orkisz, 2009, Salem et al., 2007). The direction of the axon is aligned with the direction of the minor eigenvector (the one corresponding to the smallest change in curvature: *λ*_1_) (Eberly et al., 1994). In step 3, correction is performed to keep the tracing on the medial axis of the axon, assuring the most reliable tracing. This step involves extracting a 2D plane normal to the direction of the axon and searching for the pixel with the highest intensity, which is found on the centerline of the axon because of the smoothing with a Gaussian operation. This step brings significant improvements, because the data are noisy and the Hessian matrix can provide only approximations of the change in curvature.

Axon tracing data were saved in .mat format. The tracing algorithm is freely available to researchers online (https://github.com/iocalangiu/trace-3d).

### *In Vitro* Electrophysiology and Optogenetics

Acute slice preparations were prepared 4 to 6 weeks after AAV-ChR2 injection. Coronal slices containing the AC region were checked for unilateral expression of ChR2 and IC cells were targeted in the ipsilateral and contra-DCIC (Figure 1D). The DCIC was easily identifiable along the midline, whereas the exact location of the external and central nuclei of the IC can vary greatly within the sagittal plane. Coronal brain slices were cut at a thickness of 250 μm (Campden Instruments) and immediately transferred to a holding chamber containing slicing solution composed of 85 mM NaCl, 2.5 mM KCl, 1 mM CaCl_2_, 4 mM MgCl, 1.25 mM NaH_2_PO_4_, 26 mM NaHCO_3_, 75 mM sucrose, and 25 mM glucose (pH 7.4) when bubbled with 95% O_2_ and 5% CO_2_ at 37°C. The slicing solution was then exchanged for a recording solution composed of 125 mM NaCl, 2.5 mM KCl, 2 mM CaCl_2_, 1 mM MgCl, 1.25 mM NaH_2_PO4, 26 mM NaHCO_3_, and 11 mM glucose [pH 7.4] when bubbled with 95% O_2_/5% CO_2_. IC neurons were visualized on a fixed-stage upright microscope (Olympus BX51W1) fitted with a water-immersion objective (Olympus, 63×) and a near-infrared charge-coupled camera (C7500-51, Hamamatsu CCD camera). Patch pipettes were fabricated using a two-step vertical puller (PC-10, Narishige) and thick-walled borosilicate glass capillaries (1.5 mm outer diameter [o.d.], 0.86 mm inner diameter [i.d.], Harvard Apparatus). The tip resistance was 3–5 MΩ when backfilled with the internal solution (140 mM CsCl, 4 mM NaCl, 0.5 mM CaCl_2_, 10 mM HEPES, 5 mM EGTA, and 2 mM Mg-ATP [pH 7.3], adjusted with CsOH). The current output from a Multiclamp 700B amplifier (Molecular Devices) was low-pass filtered at 10 kHz and digitized at 20 kHz using a BNC-2120 board (National Instruments), running WINWCP or WINEDR software (John Dempster, University of Strathclyde, UK). Total membrane capacitance (C_m_) was calculated from C_m_ = Q/ΔV, where Q was the charge transfer during a hyperpolarizing 10 mV step of the command voltage (ΔV). The total membrane conductance (G_m_) was calculated from G_m_ = I_ss_/ΔV, where I_ss_ was the average steady-state current during the ΔV. The electrode-to-cell series resistance (R_s_) was calculated from the relationship R_s_ = ΔV/I_p_, where I_p_ was the peak of the capacitive current transient and recordings were excluded if R_s_ increased by >30%. A blue (470 nm) collimated light-emitting diode (LED) (M470L3-C1, Thorlabs) was used for optogenetics mounted to the back of the microscope and focused through the objective lens. In a set of preliminary experiments, various lengths of blue light stimulation and intensity were used to ensure that the maximum number of AC synapses were recruited. Preliminary results demonstrated that there was no correlation between stimulus power and peak ChR2 response at LED trigger outputs of >1 V. Similarly, for stimuli lengths of 1, 2, and 3 ms, there was no correlation between stimulus length and peak ChR2 response. The optical power emitted by our 63× water immersion lens increased linearly to a maximum power of 70 μW/mm^2^ at our chosen light stimuli, giving rise to a transient response that peaked at 40 μW/mm^2^, with a 10%–90% rise time of 0.73 ms and a decay constant of 9.65 ms.

### Data Analysis and Statistical Methods

Traced neurons, axons, and synapses were quantified using customized scripts written in MATLAB. Electrophysiological data were analyzed using software provided by John Dempster (Strathclyde University, UK) and MATLAB. Data analysis was performed in two stages: initial anatomical and electrophysiological quantification was conducted independently by J.H.S. (mGRASP), I.C. (axon tracing), and D.L. (evoked and mEPSCs). Gaussian fitting of mEPSC amplitudes and mGRASP puncta surface area was performed by S.G.B., who was blinded to the identity of datasets. All-point histograms were constructed from mEPSC peak amplitude values and mGRASP surface area measurements, and these distributions were fitted with the sum of one to four Gaussians. χ^2^ values obtained from least-squares fitting were used to determine any improvement in the fit with increasing numbers of Gaussians. The Friedman-Diaconis rule (Freedman and Diaconis, 1981) was first used to optimize bin widths: $bin\;width=2IQR\left(x\right)/n^{1/3}$, where *IQR* is the interquartile range of the data and *n* is the number of observations in the dataset *x*. Least-squared fitting of multiple Gaussian functions of the type

$$y=y_{0}\frac{A}{\sqrt{\pi /2}}e^{-2\frac{{\left(x-x_{c}\right)}^{2}}{w^{2}}}$$

were used, where *y*_0_ is the offset, *A* is the area, *w* is the width, and *x*_*c*_ is the center of the Gaussian. All data are presented as mean ± SEM unless otherwise stated.

## Supplemental Information

Document S1. Figure S1

Document S2. Article plus Supplemental Information
