# Supplementary material for: Combining mGRASP and Optogenetics Enables High-Resolution Functional Mapping of Descending Cortical Projections
Source: Cell Rep. 2018 Jul 24;24(4):1071–80. doi: 10.1016/j.celrep.2018.06.076 (PMC6083038; doi:10.1016/j.celrep.2018.06.076)
Supplement: Document S1. Figure S1 [file mmc1.pdf]

Cell Reports, Volume *24*

Supplemental Information

Combining mGRASP and Optogenetics Enables High-Resolution Functional Mapping of Descending Cortical Projections

Jun Ho Song, Diana Lucaci, Ioana Calangiu, Matthew T.C. Brown, Jin Sung Park, Jinhyun Kim, Stephen G. Brickley, and Paul Chadderton

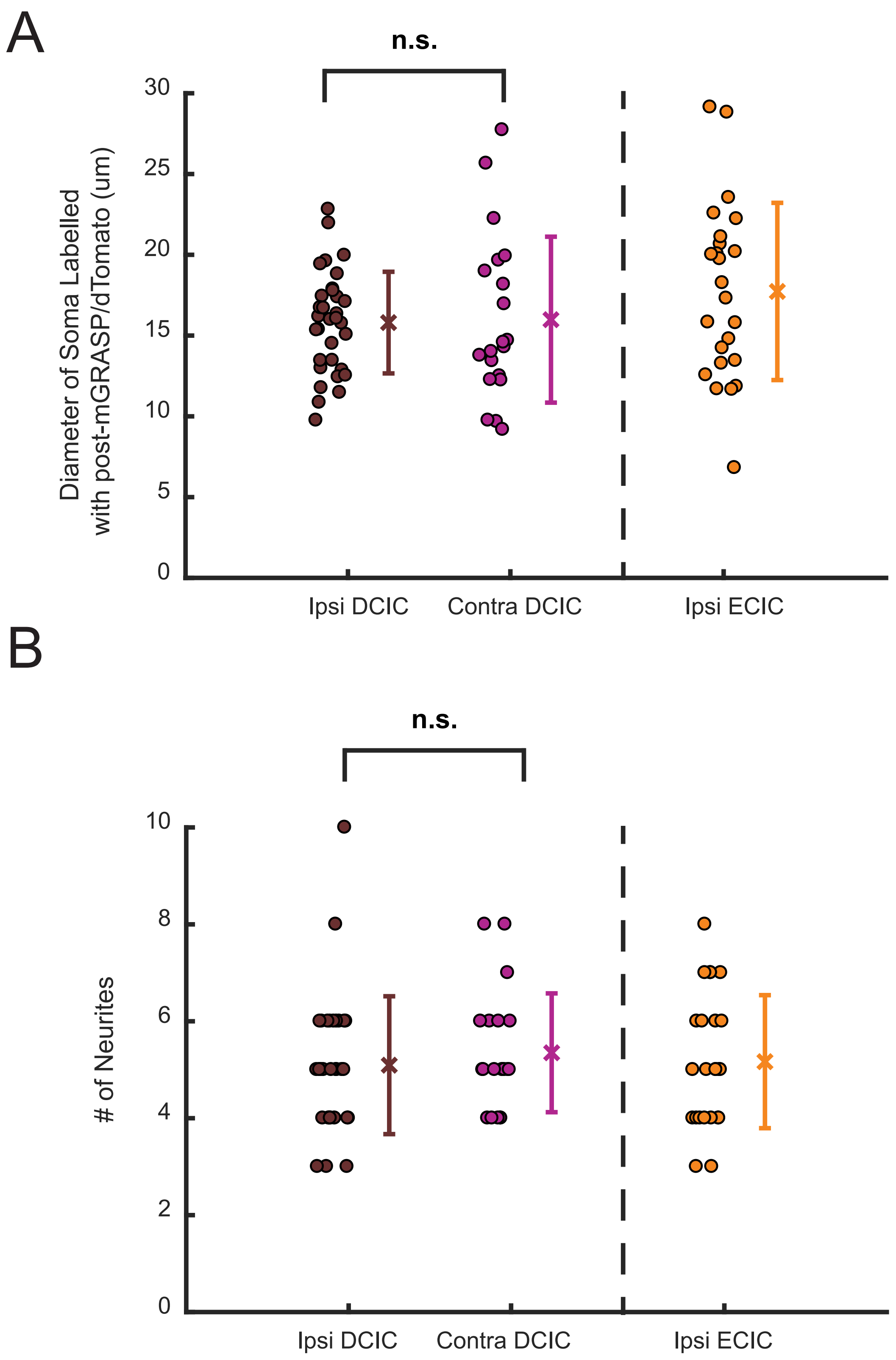

Figure S1 Morphological properties of reconstructed post-mGRASP/dTomato-expressing IC neurons. Soma diameter (**A**) and neurite number (**B**) for all reconstructed neurons in dorsal and external cortex of IC. Related to **Figure 3**.
